# Supplementary figures and images for: Susceptibility of flexible plastic foodstuffs packaging against Monomorium indicum (Hymenoptera: Formicidae) household ants
Source: PeerJ. 2024 Feb 29;12:e16782. doi: 10.7717/peerj.16782 (PMC10909368; doi:10.7717/peerj.16782)

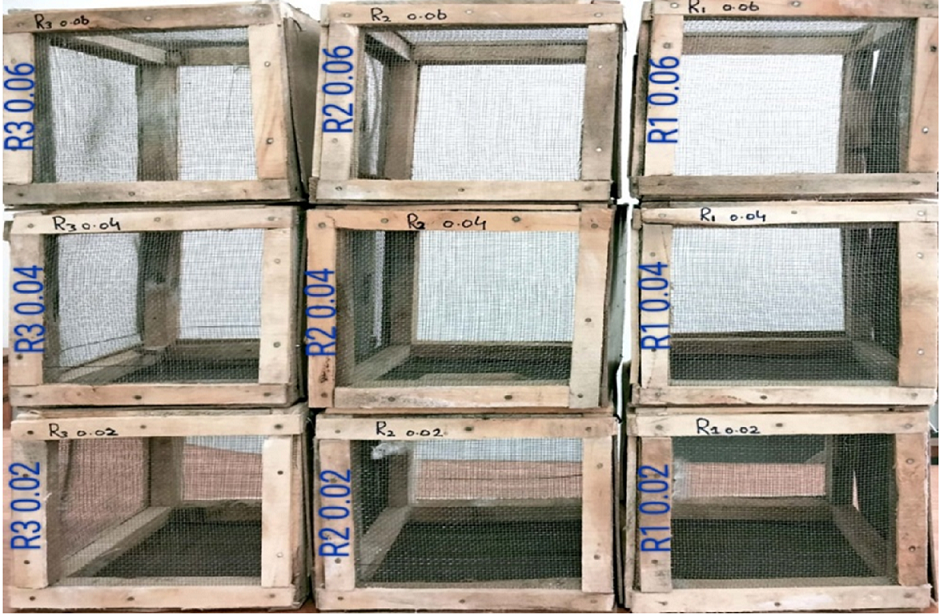

Supplement: Figure S1 [file peerj-12-16782-s001.png]

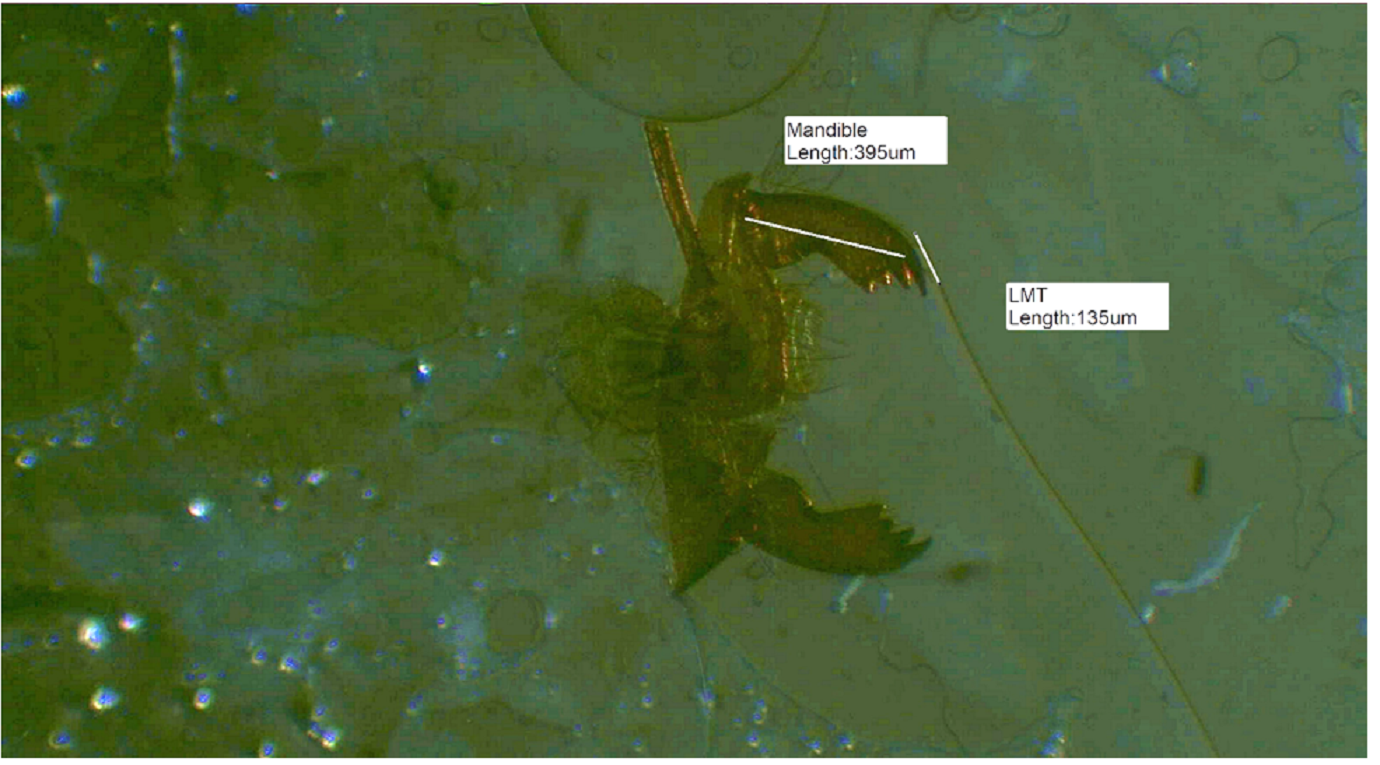

Supplement: Figure S2 [file peerj-12-16782-s002.png]

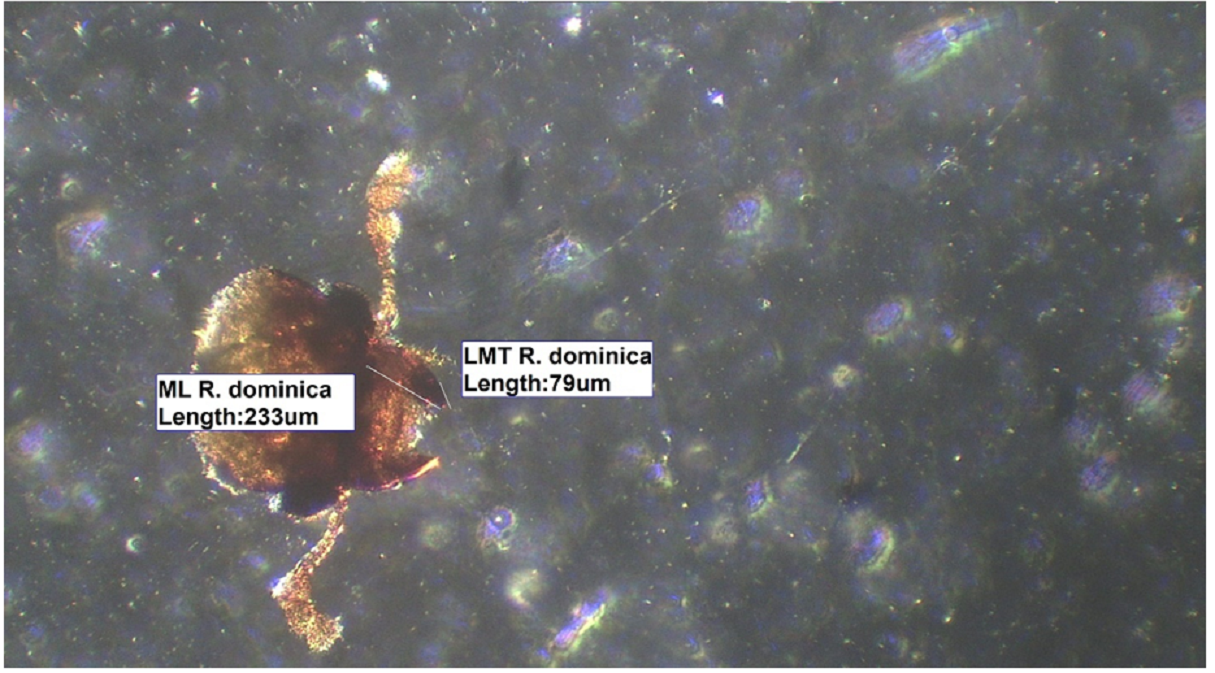

Supplement: Figure S3 [file peerj-12-16782-s003.png]

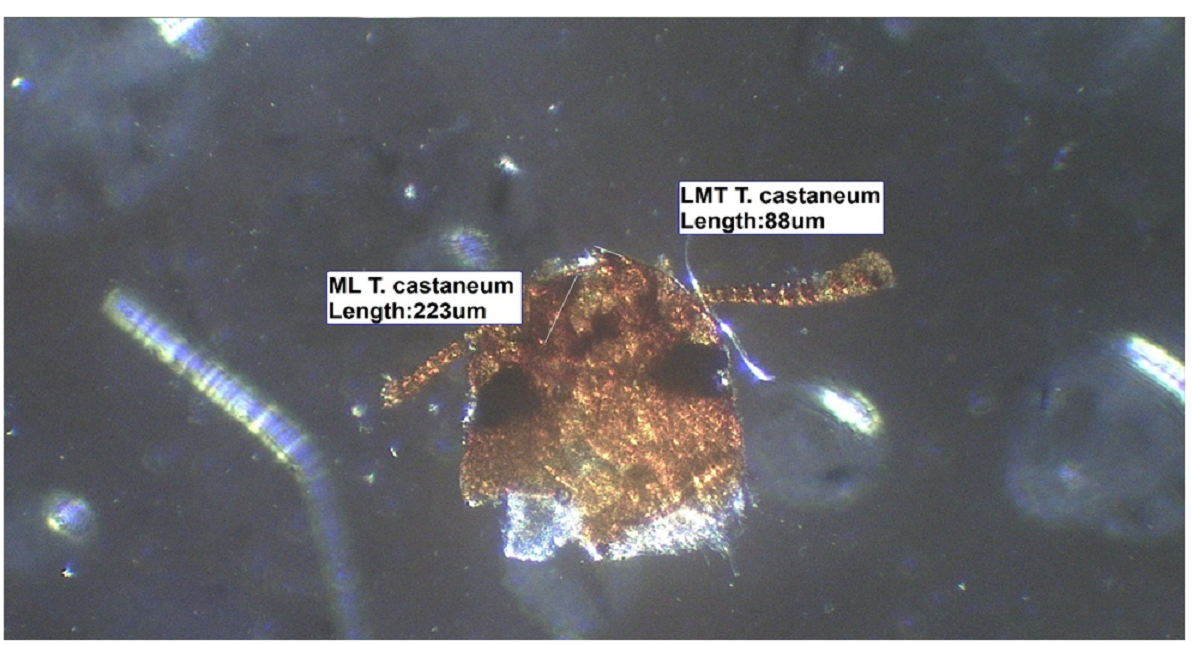

Supplement: Figure S4 [file peerj-12-16782-s004.png]

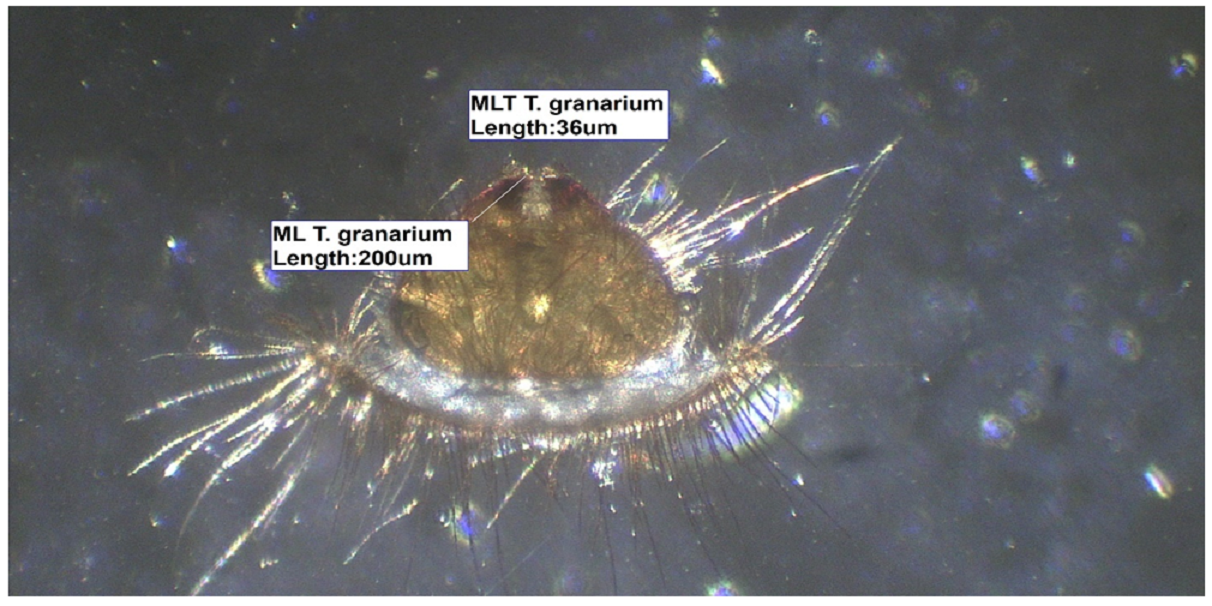

Supplement: Figure S5 [file peerj-12-16782-s005.png]
